# Cathepsin Inhibition-Induced Lysosomal Dysfunction Enhances Pancreatic Beta-Cell Apoptosis in High Glucose

**DOI:** 10.1371/journal.pone.0116972

**Published:** 2015-01-27

**Authors:** Minjeong Jung, Jaemeun Lee, Hye-Young Seo, Ji Sun Lim, Eun-Kyoung Kim

**Affiliations:** 1 Department of Brain Science, Daegu Gyeongbuk Institute of Science & Technology, Daegu, Korea; 2 Neurometabolomics Research Center, Daegu Gyeongbuk Institute of Science & Technology, Daegu, Korea; IISER-TVM, INDIA

## Abstract

Autophagy is a lysosomal degradative pathway that plays an important role in maintaining cellular homeostasis. We previously showed that the inhibition of autophagy causes pancreatic β-cell apoptosis, suggesting that autophagy is a protective mechanism for the survival of pancreatic β-cells. The current study demonstrates that treatment with inhibitors and knockdown of the lysosomal cysteine proteases such as cathepsins B and L impair autophagy, enhancing the caspase-dependent apoptosis of INS-1 cells and islets upon exposure to high concentration of glucose. Interestingly, treatment with cathepsin B and L inhibitors prevented the proteolytic processing of cathepsins B, D and L, as evidenced by gradual accumulation of the respective pro-forms. Of note, inhibition of aspartic cathepsins had no effect on autophagy and cell viability, suggesting the selective role of cathepsins B and L in the regulation of β-cell autophagy and apoptosis. Lysosomal localization of accumulated pro-cathepsins in the presence of cathepsin B and L inhibitors was verified via immunocytochemistry and lysosomal fractionation. Lysotracker staining indicated that cathepsin B and L inhibitors led to the formation of severely enlarged lysosomes in a time-dependent manner. The abnormal accumulation of pro-cathepsins following treatment with inhibitors of cathepsins B and L suppressed normal lysosomal degradation and the processing of lysosomal enzymes, leading to lysosomal dysfunction. Collectively, our findings suggest that cathepsin defects following the inhibition of cathepsin B and L result in lysosomal dysfunction and consequent cell death in pancreatic β-cells.

## Introduction

The integrity of pancreatic β-cell function and mass is critical for the pathogenesis of diabetes [1]. Although glucose is the main regulator of insulin biosynthesis and secretion, chronic hyperglycemia is associated with impaired function of insulin secretion. The detrimental effect of excessive glucose concentration is referred to as 'glucotoxicity' [2,3], which can negatively affect β-cell mass by inducing apoptosis [4]. Glucotoxicity is associated with the induction of endoplasmic reticulum (ER) stress, mitochondrial dysfunction and oxidative damage to proteins [5,6].

Mounting evidence has indicated that autophagy plays an important role in cell survival and death in response to cellular stress. Under certain stress conditions, autophagy can protect cells against cytotoxicity [7,8]. For example, it provides a protective role by removing cellular components damaged by oxidative stress [9–11]. Autophagy is a dynamic process associated with the formation of autophagosomes, double-membrane vacuoles that engulf cellular components. The autophagosomes subsequently fuse with lysosomes to form autolysosomes, which degrade the dysfunctional cytoplasmic organelles and damaged proteins using lysosomal hydrolytic enzymes [12]. Therefore, autophagy maintains tissue homeostasis and ensures cell survival under stress conditions. [13–17]. Dysregulation of autophagy has been indicated in the pathogenesis of several diseases including neurodegenerative disease, heart disease, cancer and aging [7,18–20].

Microtubule-associated protein light-chain 3 (LC3), also called autophagy-related protein 8 (Atg8) in yeast, is processed to LC3-I, and then conjugated with phosphatidylethanolamine (PE) through the mediation of the Atg5/Atg12 complex to generate membrane-associated LC3-II [21–23]. LC3-II stays on the membrane until it is degraded by the lysosome, thus it is widely used as a marker for autophagic process [18].

The progression and resolution of autophagy critically depends on lysosomal function, as lysosomes play a role in the degradation of cellular compartments. Lysosomes contain many types of hydrolytic enzymes, such as peptidases, phosphatase, nucleases, glycosidases, protease and lipase, which can digest most macromolecules in the cell [24]. Cathepsins represent a major class of lysosomal proteases, especially important for the execution of autophagy [25–27]. The cathepsin family consists of aspartic, cysteine, and serine cathepsins. Aspartic cathepsins include cathepsin D and E, while cysteine cathepsins include cathepsin B, C, H, K, and L, and cathepsin A and G belong to serine cathepsins [25].

Cathepsins are synthesized as inactive (immature) pro-cathepsins and are proteolytically processed to form active (mature) cathepsins [28,29]. They contain a signal peptide which is cleaved within the ER, and are then transported into the endosome/lysosome compartment via mannose-6-phosphate receptors. Most lysosomal cathepsins are functionally optimized at low pH, as cathepsins are stable and active at acidic pH.

Recent studies have shown that autophagy is associated with diabetes through its effects on pancreatic β-cells [30–32]. We previously reported that dysregulation of autophagy causes apoptotic cell death, suggesting that autophagy plays a protective role in the survival of pancreatic β-cells [33]. In this study, we investigate the mechanism by which inhibition of aspartic and cysteine cathepsins results in lysosomal dysfunction, enhancing pancreatic β-cell apoptosis in conditions of high glucose.

## Materials and Methods

### Antibodies and chemical reagents

Antibodies against cleaved caspase-3, cleaved caspase-9, Bcl-2, phosphor-JNK (Thr183/Tyr185), JNK and GAPDH were obtained from Cell signaling. Antibodies against poly ADP ribose polymerase (PARP) were purchased from BD Biosciences, and those against LC3 and lysosomal-associated membrane protein 2 (LAMP2) were from Sigma. Antibodies against cathepsin L and cathepsin D were purchased from Santa Cruz, while cathepsin B was from Millipore. Cathepsin B (CA074), K (Z-L-NHNHCONHNH-LF-Boc, II), and L (Z-FY(t-Bu)-DMK, III) inhibitors, along with E64d were purchased from Calbiochem. Pepstatin A and SP600125 (JNK inhibitor) were purchased from Sigma.

### Cell culture

Rat insulinoma β-cell line INS-1 (832/13) [34] (generously provided by Dr. Christopher Newgard, Department of Pharmacology and Cancer Biology, Duke University Medical Center, Durham, NC, U.S.A) and a stable INS-1 cell line (GFP-LC3/INS-1) expressing GFP-LC3 from INS-1 cells [35] were cultured in a 37°C incubator with 5% CO_2_ in RPMI 1640 medium (GIBCO) supplemented with 10% fetal bovine serum (Hyclone), 11 mM glucose (Sigma), 2 mM L-glutamine (GIBCO), 10mM 4-2-hydroxyethyl-1-piperazineethanesulfonic acid (HEPES) (GIBCO), 1 mM sodium pyruvate (GIBCO), 0.05 mM 2-mercaptoethanol (GIBCO), and 1% penicillin-streptomycin (Hyclone)._._

### Cell viability assay

To assess cell viability, INS-1 cells were seeded in 96-well plates at a density of 4 × 10^5^ cells per mL in 11 mM glucose or 30 mM glucose medium treated with lysosomal protease inhibitors for the indicated times. After CellTiter-Blue (Promega) was added to each well and incubated for 3 hr, the absorbance of the samples was measured with a microplate reader at 560 nm excitation and 590 nm emission.

### Annexin V staining

INS-1 cells were plated in 12-well plates at a density of 6.5 × 10^5^ cells per mL in 11 mM glucose medium treated with E64d, cathepsin B and L inhibitors, and staurosporine. Cells were stained with incubation buffer containing Annexin V and Hoechst 33342 (Invitrogen) for 15 min at 37°C after removal of the medium. Apoptotic cells were assessed using an Alexa Fluor® 488 Annexin V/Dead Cell Apoptosis Kit (Invitrogen) according to the manufacturer's protocol. Images of the apoptotic cells were obtained with a fluorescence microscope (Zeiss).

### Immunoblot analysis

INS-1 cells were plated in 6-well plates at a density of 6.5 × 10^5^ cells per mL in 11 mM glucose or 30 mM glucose medium treated with E64d, and cathepsin B and L inhibitors. Samples were subjected to 10% SDS-polyacrylamide gel electrophoresis and subsequently transferred onto a polyvinylidene difluoride (PVDF) membrane. After transfer, the membrane was incubated with the specific primary antibodies at 4°C overnight. After three washes with Tris-Buffered Saline and Tween 20 (TBST) buffer, the membrane was incubated with horseradish peroxidase (HRP)-linked secondary antibody and visualized by Supersignal West Pico Chemiluminescent Substrate (Thermo), according to the recommended procedure.

### Pancreatic islet culture

Rat islets were isolated as previously described [32] from adult male Sprague Dawley (SD) rats (200–250g). Animal experimentation was done in accordance with the guidelines on care and use as approved by the DGST institutional Animal Care and Use Committee. Islets were cultured in INS-1 medium containing 11 or 30 mM glucose, treated with cathepsin B and L inhibitors.

### Immunofluorescence analysis

INS-1 or GFP-LC3/INS-1 cells seeded on coverslips were cultured in 11 mM or 30 mM glucose medium with E64d, and cathepsin B and L inhibitors. For Lysotracker staining, the cells were first stained with 300 nM Lysotracker Red DND-99 (Invitrogen) for 10 min at room temperature (RT). After staining, the cells were fixed with 4% formaldehyde for 10 min and then incubated in PBS with 0.1% TritonX-100, 0.1 M glycine for 15 min at RT for permeabilization. The cells were washed with PBS, and blocked for 10 min at RT. Cells were incubated with anti-cathepsin B (Millipore), anti-cathepsin L (Santa Cruz) and anti-PDI (Abcam) for 3 hr at RT. Cells were incubated for an additional 3 hr at RT with secondary antibodies, and then stained with Hoechst 33342 (Invitrogen) for 10 min at RT. Coverslips were mounted with ProLong Gold antifade reagent (Invitrogen). The cells treated with E64d and cathepsin B and L inhibitors on coverslips were analyzed with an LSM700 Confocal microscope (Zeiss). The images were analyzed using the ZEN2009 software. The data illustrated were from one representative experiment of at least three independent repeats.

### Cathepsin activity

INS-1 cells were plated in 6-well plates at a density of 6.5 × 10^5^ cells per mL in 30 mM glucose medium in presence or absence of cathepsin inhibitors for 24, 48 and 72 hr. The activity of cathepsin B and L was assessed using the Cathepsin B and L activity kit (Calbiochem), as recommended by the manufacturer.

### siRNA knock-down for cathepsin B and L

ON-TARGET plus SMARTpool (DHARMACON) composed of four different siRNAs against cathepsin B or L were used to obtain the higher knock-down efficiency and to reduce the off-target effects. siRNAs treatments were carried out on INS-1 cells seeded in 12-well plates containing 30 mM glucose medium using Lipofectamine 2000. The protein extracts were prepared at 48 and 72 hr post-transfection.

### Nuclear and cytoplasmic fractionation

Cytoplasmic and nuclear fractions were prepared using a kit purchased from Thermo. INS-1 cells were harvested with trypsin-EDTA, centrifuged at 500 × g for 5 min, and washed by suspending the cell pellet with PBS. The cells were transferred to a 1.5 mL microcentrifuge tube and then centrifuged at 500 × g for 2–3 min. After buffers were added to the cell pellet and centrifuged, the supernatants (cytoplasmic extract) were transferred to a clean pre-chilled tube. The pellet, containing the nuclei, was suspended in ice-cold buffer. The pellet was centrifuged, and then the supernatants (nuclear extract) were transferred to a clean pre-chilled tube. Protein concentrations of the two fractions were assayed using the Bicinchoninic Acid (BCA) assay kit (Thermo).

### Enriched lysosomal extraction

Extraction for lysosomal enrichment was carried out with a kit (PIERCE). INS-1 cells were harvested and centrifuged at 850 × g for 2 min. After removal of the supernatants, the pellet was resuspended with Lysosome Enrichment Reagent A and placed on ice for 2 min. The cells were broken with a Dounce tissue grinder on ice, after which Lysosome Enrichment Reagent B was added. The cells were then centrifuged at 500 × g for 10 min at 4°C, and the supernatants were overlaid on the density gradients and further subjected to ultracentrifugation at 145,000 × g for 2 hr at 4°C. After ultracentrifugation, the lysosome band located at the top of the gradient was carefully removed. The samples were diluted with 2–3 volumes of PBS, and were centrifuged at 18,000 × g for 30 min at 4°C. The lysosome pellets were stored on ice until used. The lysosome pellets were then boiled with SDS-PAGE sample buffer and analyzed with western blotting.

### Transfection of mRFP-GFP-LC3 constructs

INS-1 cells cultured in 30 mM glucose were seeded on cover glasses in 6-well plates and transfected with mRFP-GFP-LC3 plasmids (a gift from Dr. Mook Inhee, Seoul National University) using Lipofectamine 2000 (Invitrogen). Transfected INS-1 cells were treated daily with cathepsin B and L inhibitors for 48 hr. The cells were fixed with 4% formaldehyde, and the coverslips were then mounted with ProLong Gold antifade reagent (Invitrogen). The images were analyzed with an LSM700 Confocal microscope (Zeiss).

### Statistical analysis

All results were represented as mean ± SEM. When comparisons between groups were required, statistical significance was determined by an unpaired *t* test. *P* values of <0.05 were considered statistically significant.

## Results

### Inhibition of cathepsin B and L increases β-cell apoptosis

**Figure 1:**
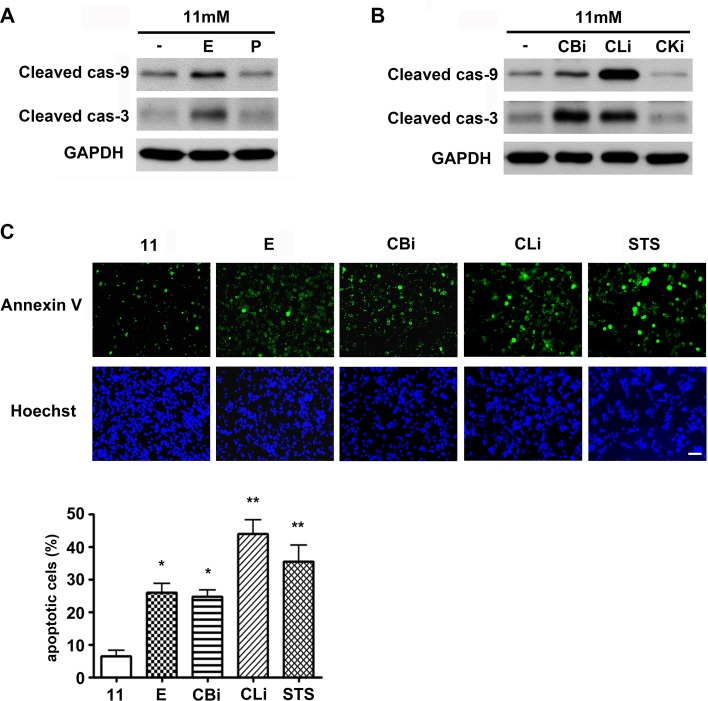
Inhibition of cathepsins B and L triggers apoptosis in INS-1 cells. (A) INS-1 cells were treated with E64d (E, 20 μg/mL) and pepstatin A (P, 20 μg/mL) in 11 mM glucose medium for 48 hr, and caspase activation was monitored by western blotting using antibodies detecting cleaved, active forms of caspases. (B) INS-1 cells were treated with cathepsin B inhibitor (CBi, 20 μM), cathepsin L inhibitor (CLi, 20 μM), and cathepsin K inhibitor (CKi, 20 μM) for 48 hr in 11 mM glucose medium, and caspase activation was monitored by western blotting. (C) INS-1 cells stained with Annexin-V and Hoechst were observed using a fluorescence microscope for quantification of apoptotic cells. Cells were treated with E, CBi, CLi and staurosporine (STS, 0.5 μM) in 11 mM glucose medium. Lysosomal protease inhibitors were added daily, and STS was added 6 hr prior to staining (three independent counts). STS is a known prototypic inducer of apoptosis. *p <0.01, ** *p* <0.05 compared with 11 mM glucose. The scale bar represents 20 μm.

In this study, the effect of pharmacological inhibition of lysosomal proteases on β-cell death was examined. INS-1 cells were cultured in the presence of lysosomal protease inhibitors in 11 mM glucose (normal culture concentration) medium for 48 hr, and then the activation of apoptosis was analyzed by western blotting with antibodies against the active, cleaved form of caspases (Fig. 1A). Among the inhibitors of lysosomal proteases, E64d has inhibitory effects on cysteine proteases such as cathepsin B, K, and L, whereas pepstatin A has inhibitory effects on aspartic proteases such as cathepsin D and E. E64d treatment was observed to increase caspase-9 and -3 activation, whereas pepstatin A had no effect on caspase activation (Fig. 1A). Among the lysosomal cysteine proteases inhibitors, inhibitors of cathepsin B and L, but not cathepsin K, also increased caspase-9 and -3 activation (Fig. 1B). These results demonstrated that the inhibition of cathepsin B and L specifically triggers caspase-dependent apoptosis. The consequent increase in apoptotic cell death was next confirmed using Annexin-V staining, following treatment with lysosomal cysteine protease inhibitors. INS-1 cells treated with staurosporine (STS), a known prototypic inducer of apoptosis, were used as a positive control. Increases in cell death by E64d, cathepsin B and L inhibitors were represented by increasing numbers of Annexin V-positive cells (Fig. 1C). Compared to the 11 mM glucose control, apoptotic cell death was significantly increased by 4.0, 3.9, and 6.9 fold following treatment with E64d, cathepsin B and L inhibitors, respectively. These results suggest that cathepsin B and/or L play an important role in β-cell survival, as inhibition of their activity increases caspase-dependent apoptosis.

### Inhibition of cathepsin B and L enhances cell death under glucotoxicity

The detrimental effects of glucotoxicity negatively affect β-cell mass by apoptosis [36]. In order to investigate whether the inhibition of lysosomal proteases worsens β-cell apoptosis associated with glucotoxicity, hyperglycemic conditions were manipulated by culturing INS-1 cells in 30 mM glucose medium, which has been shown to decrease insulin mRNA levels [37] and induce ER stress in INS-1 cells by glucotoxicity [38].

**Figure 2:**
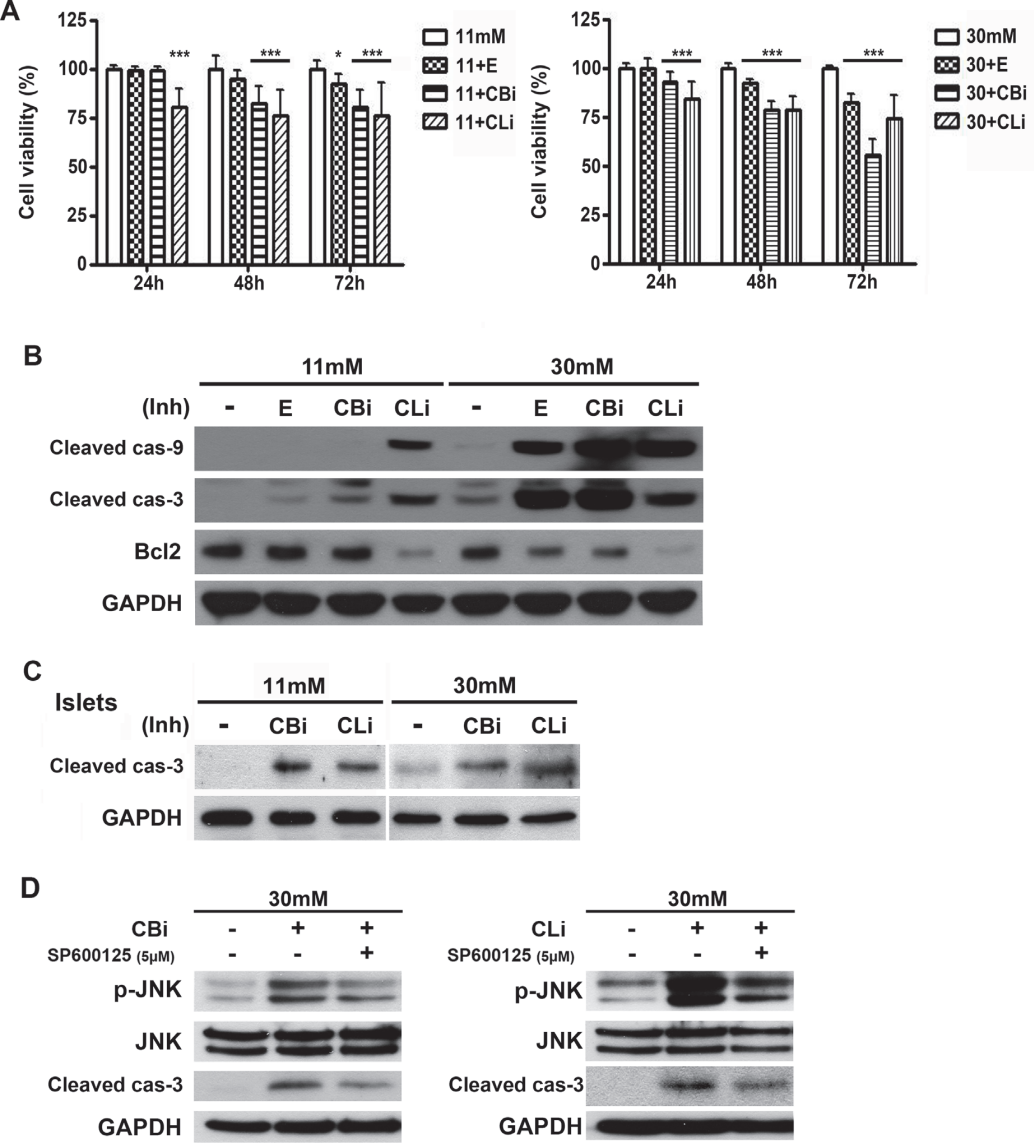
Inhibition of cathepsins B and L increases caspase-dependent apoptosis under glucotoxicity. (A) INS-1 cells were treated with E64d (E, 20 μg/mL), cathepsin B inhibitor (CBi, 20 μM), and cathepsin L inhibitor (CLi, 20 μM) in INS-1 medium containing 11 mM or 30 mM glucose. Cell viability was assessed using the CellTiter-BlueⓇ Cell Viability assay and indicated as a percentage of the values measured at each time point (n = 15). Statistical significance was represented as * *p* <0.01, and *** *p* <0.001 compared with 11 mM glucose and 30 mM glucose. (B) Protein levels of active caspase-9 (cleaved cas-9), caspase-3 (cleaved cas-3) and Bcl-2 were measured in INS-1 cells cultured in 11 or 30 mM glucose medium for 48 hr. Cells were treated daily with E, CBi, and CLi. (C) Immunoblot analysis of pancreatic islets of SD rats treated with CBi and CLi in 11 mM or 30 mM glucose medium for 48 hr. (D) JNK inhibitor (SP600125, 5 μM) was treated with CBi and CLi in INS-1 cells cultured in 30 mM glucose medium for 24 hr.

To assess the effects of inhibition of cysteine cathepsins on INS-1 cell survival in 30 mM glucose medium, cell viability was compared in 11 mM and 30 mM glucose conditions. Treatment with E64d and cathepsin B and L inhibitors caused a more significant decline in INS-1 cell viability in high glucose medium than in 11 mM glucose (Fig. 2A). The levels of anti-apoptitic Bcl-2 and active forms of caspase-9 and caspase-3 were analyzed at 48 hr (Fig. 2B). The results indicated that E64d, and cathepsin B and L inhibitors effectively increased the activation of caspase-9 and -3, while decreasing the protein level of Bcl-2 more prominently in the 30 mM glucose medium than in 11 mM glucose (Fig. 2B).

Pancreatic islets from Sprague-Dawley (SD) rats cultured in 30 mM glucose medium also showed an increased caspase-3 activation when treated with cathepsin B and L inhibitors (Fig. 2C).

JNK activation is one of the upstream kinases in caspase-3 dependent apoptosis pathway [39,40]. Increases in phosphorylated JNK were observed in cathepsin B or L inhibitor-treated INS-1 cells in 30 mM glucose medium at 24 hr (Fig. 2D). The specific JNK inhibitor, SP600125, reduces the cathepsin inhibitor-induced activation of caspase-3. The activated JNK might be involved in activation of caspase-3, resulting in the induction of apoptosis.

Taken together, these results indicate that cathepsin B and L inhibitors enhance caspase 3-dependent apoptosis, leading to a reduction in pancreatic β-cell survival under glucotoxicity.

### Inhibition of cathepsin B and L leads to LC3-II accumulation

**Figure 3:**
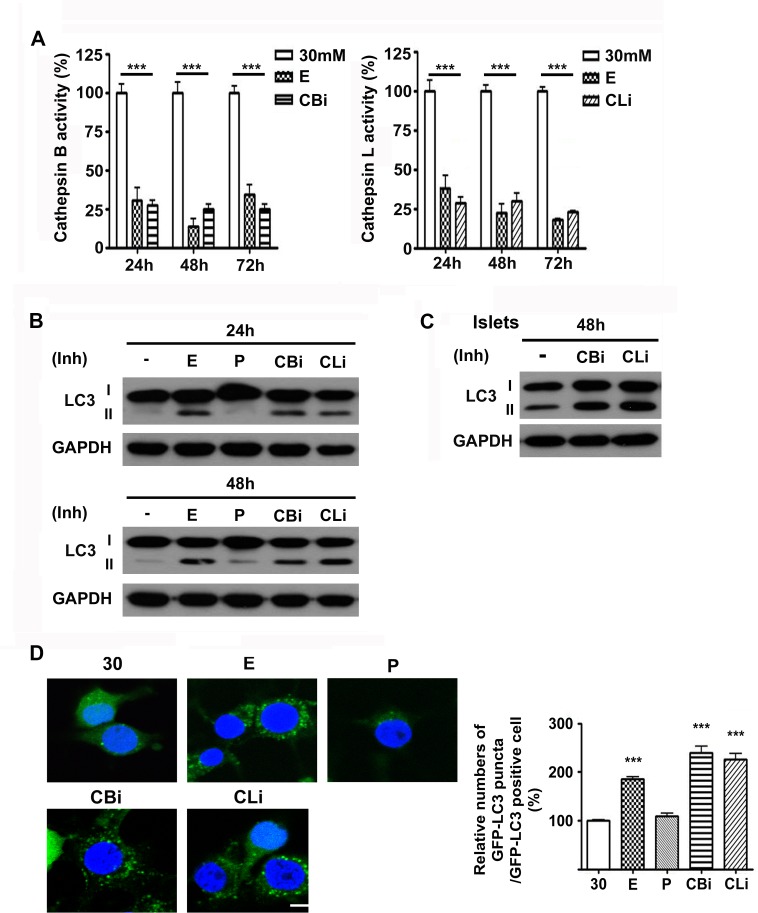
Inhibition of cathepsins B and L leads to LC3 accumulation. (A) Activity of cathepsins B and L in 30 mM glucose with E64d (E, 20 μg/mL), cathepsin B inhibitor (CBi, 20 μM) or cathepsin L inhibitor (CLi, 20 μM) was measured at 24 hr, 48 hr and 72 hr (n = 9). Statistical significance is represented as *** *p* <0.001 compared with 30 mM glucose. (B) INS-1 cells cultured in 30 mM glucose for 24 or 48 hr were treated daily with E, P, CBi, and CLi. LC3-II accumulation was assessed by immunoblot analysis. (C) Islets from SD rat cultured in 30 mM glucose and treated with CBi, and CLi. Level of LC3 was measured by immunoblot assay. (D) GFP-LC3/INS-1 stable cells were treated daily with E, P, CBi, and CLiin in 30 mM glucose for 48 hr. The graph indicates the number of GFP-LC3 puncta counted among GFP positive cells (n = 5). *** *p* <0.001 compared with 30 mM glucose. The scale bar represents 5 μm.

We further focused on the effects of lysosomal cysteine cathepsin inhibition on the autophagy process in high glucose conditions. The inhibition of cathepsins was verified by measuring their activities in the presence of the inhibitors (Fig. 3A). Cathepsin B and L activities were significantly reduced by 75% and 78% following treatment with inhibitors in 30 mM glucose, respectively (Fig. 3A). An increase in LC3-II level or LC3 puncta formation can occur due to either an increased rate of autophagy flux (“on-state”) or impaired autophagy flux accompanied by incomplete degradation of LC3 (“off-state”). When cells are treated with inhibitors of lysosomal proteases such as E64d and pepstatin A, the degradation of LC3-II is blocked, leading to accumulation of LC3-II and the off-state of autophagy [41]. In line with the impairment of autophagy following the inhibition of lysosomal cathepsins, the treatment of INS-1 cells with lysosomal cysteine protease inhibitors, including E64d, and cathepsin B and L inhibitors, resulted in the incomplete degradation of LC3 and subsequent accumulation of LC3-II at 24 and 48 hr (Fig. 3B).

The inhibition of cathepsin B and L resulted in the accumulation of LC3-II not only in INS-1 cells, but also in the islets. Islets from SD rats showed similar results when treated with cathepsin B and L inhibitors in 30 mM glucose medium. Increased accumulation of LC3-II in the islets was detected at 48 hr (Fig. 3C).

The increase in LC3 puncta formation by treatment with E64d and cathepsin B and L inhibitors is also indicative of impaired autophagy flux, as was the case of LC3-II protein level (Fig. 3D). To monitor LC3 puncta, ring-shaped or punctate green fluorescence signal was measured in a stable INS-1 cell line expressing GFP–LC3 (GFP–LC3/INS-1) cultured in 30 mM glucose in the presence or absence of inhibitors. Compared with the 30 mM glucose control, treatment of GFP–LC3/INS-1 cells with E64d, cathepsin B and L inhibitors increased the number of cells containing GFP–LC3 puncta up to 185%, 240% and 226% at 48 hr, respectively (Fig. 3D). This increase in GFP-LC3 puncta accumulation was due to the incomplete degradation of LC3 by impaired autophagy, as seen in Fig. 3B. In contrast, pepstatin A, an aspartic protease inhibitor, had no effect on LC3 accumulation (Fig. 3B). In addition, no significant changes in punctate signals in GFP-LC3/INS-1 cells treated with pepstatin A were observed (Fig. 3D). These data strongly suggest the selective role of cathepsin B and L in the regulation of pancreatic β-cell autophagy.

### Knockdown of cathepsin B and L enhances caspase-3 activation and increases LC3-II accumulation

**Figure 4:**
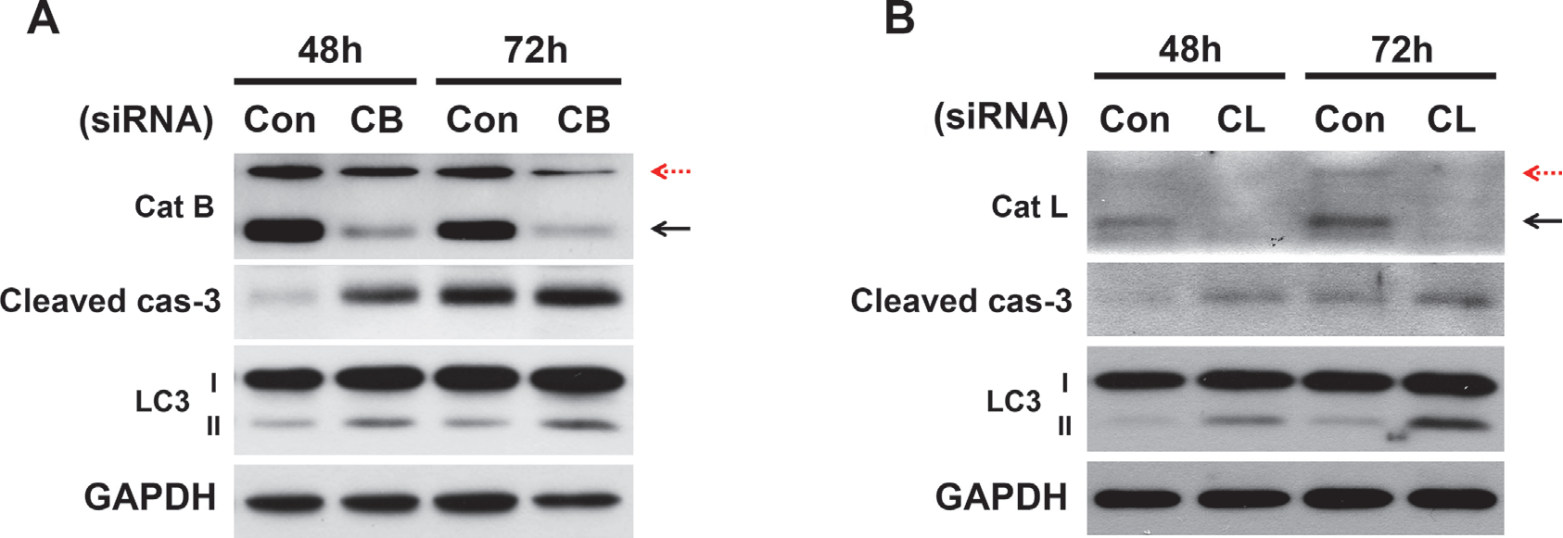
Cathepsin B- and L-knockdown with siRNA enhances apoptosis and impairs autophagy. INS-1 cells were transfected with scrambled siRNA (con) or with either the cathepsin B (A) or cathepsin L (B) siRNA and cultured in 30 mM glucose for 48 and 72 hr. Immunoblot analysis was performed at the indicated time points post-transfection. Red dashed arrows indicate immature cathepsins, while block solid arrows indicate the mature active form of cathepsins.

The inhibitor studies demonstrated the critical role of cathepsins B and L in the apoptosis and autophagy of pancreatic β-cells. To confirm the role of cathepsins B and L via a genetic method, cathepsin B or L was depleted through the use of siRNA (Fig. 4). Cathepsin B and L were knocked-down in INS-1 for 48 and 72 hr. The knockdown with siRNA in 30 mM glucose medium resulted in an increase in apoptotic cell death through caspase-3 activation. This result is consistent with the pharmacological inhibition of cathepsins, as shown in Fig. 2. In addition, the depletion of cathepsins B and L also increased LC-3 accumulation, which is consistent with the results obtained by the treatment with inhibitors in Fig. 3. In contrast, when cathepsin K was knocked down with siRNAs there were no changes in activation of caspase-3 and LC-3 accumulation as expected (data not shown).

### Inhibition of cathepsin B and L results in accumulation of pro-cathepsins in the lysosomes

**Figure 5:**
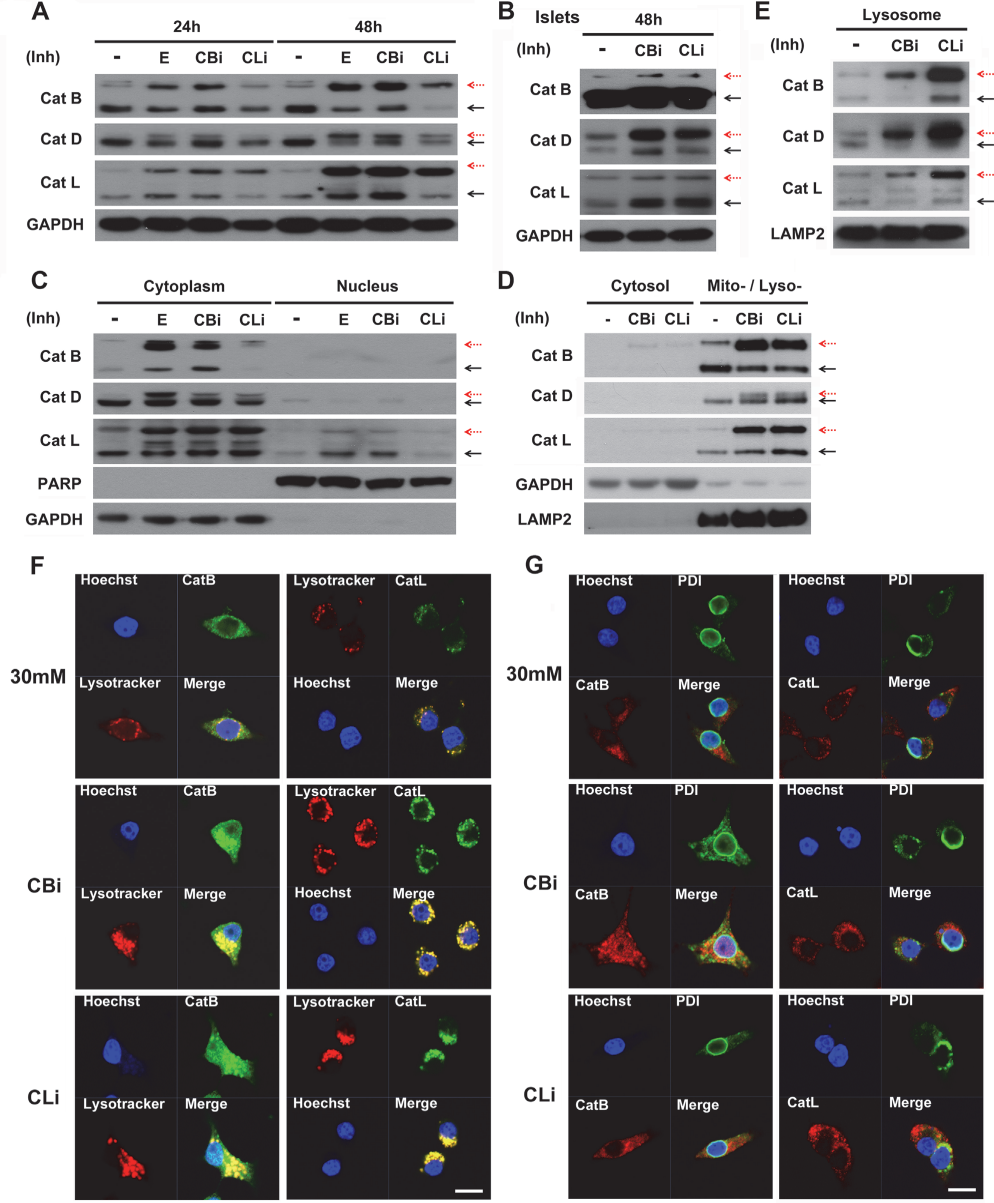
Inhibition of cathepsins B and L results in incomplete processing of cathepsins in lysosome, not in ER. (A) Immunoblot analysis with E64d (E, 20 μg/mL), cathepsin B inhibitor (CBi, 20 μM), and cathepsin L inhibitor (CLi, 20 μM)-treated INS-1 cells in 30 mM glucose for 24 or 48 hr. (B) Immunoblot analysis of pancreatic islets of SD rats treated daily with CBi and CLi in 30 mM glucose for 48 hr. (C) Cytoplasmic and nuclear fractions were prepared from INS-1 cells cultured in 30 mM glucose medium with cathepsin B and L inhibitors for 48 hr then immunoblotted. (D) Localization of pro-cathepsins and cathepsins after treatment with cathepsin B and L inhibitors in 30 mM glucose for 48 hr by cytosol and mito- / lyso- (mitochondria / lysosomal) fractionation. (E) Lysosome-enrichment extraction was prepared and separated by 10% SDS-PAGE. Fraction purity and loading were controlled by immunoblotting for LAMP2 (a lysosomal marker). Red dashed arrows indicate immature cathepsins, while block solid arrows indicate mature cathepsins. (F) Co-localization of cathepsin B (CatB) and L (CatL) with lysotracker was observed in INS-1 cells treated daily with CBi and CLi in 30 mM glucose for 48 hr, as detected by immunofluorescence analysis. (G) Co-localization of CatB / CatL and protein disulfide isomerase (PDI) observed in INS-1 cells treated daily with CBi and CLi in 30 mM glucose for 48 hr. Nuclei were stained with Hoechst 33342 dye. The scale bar represents 5 μm.

Studies have shown that cathepsins are involved in the processing of other cathepsins [42–46]. To examine whether the inhibition of cathepsin B and L has an effect on the processing of other cathepsins, changes in the forms of cathepsins were assessed by immunoblot analysis in INS-1 cells and pancreatic islets in 30 mM glucose (Fig. 5A and 5B).

As shown in Fig. 5A, the proteolytic processing of both cysteine proteases (cathepsins B and L) and aspartic proteases (cathepsin D) were inhibited by the cathepsin B and L inhibitors. The inhibitors of cathepsin B and L led to the accumulation of pro-cathepsin B, L and D, as denoted by a red dashed arrow. Pro-cathepsin B, L and D exhibited greater accumulation at 48 hr than 24 hr in INS-1 cells.

Similarly, the inhibition of cathepsins B and L showed incomplete processing of the cathepsins in the pancreatic islets of SD rats (Fig. 5B).

To determine the subcellular localization of the accumulated pro-cathepsins, the cytoplasmic (non-nuclear) and nuclear fractions were first analyzed. Both pro-cathepsins and cathepsins (B, D and L) were found to be predominantly localized in the cytoplasmic fraction in the presence of cathepsin B and L inhibitors (Fig. 5C). Next, the cytoplasmic fraction was further separated to assess the levels of pro-cathepsin accumulation in the cytosolic and mitochondrial/lysosomal fractions (Fig. 5D). Much higher accumulation of the pro-cathepsins was observed in the mitochondrial/lysosomal fractions compared with the cytosolic fraction. The lysosomal localization of cathepsins was further verified using a lysosomal enrichment kit. The enriched lysosomal fraction contained a high accumulation of pro-cathepsins when treated with inhibitors (Fig. 5E). In addition, fluorescence imaging by staining with Lysotracker, a lysosome marker, showed co-localization of the lysosomes and cathepsins in 30 mM glucose culture conditions, which was further increased upon treatment with cathepsin B or L inhibitors (Fig. 5F).

Cathepsins become mature by cleavage of the N-terminal signal peptide within the ER, and are then activated through proteolytic processing in the endosome and lysosome [47–53]. To observe whether the pro-cathepsins accumulated in the ER after treatment with cathepsin B and L inhibitors, the co-localization of cathepsin B/L and protein disulfide isomerase (PDI), a known ER marker, was examined using immunofluoresence (Fig. 5G). The results indicated that PDI and cathepsin B/L do not co-localize in the ER following the inhibition of cathepsins B and L, which is in sharp contrast to the substantial co-localization of cathepsin with the lysosomes.

Taken together, these findings indicate that the inhibition of cathepsins B and L blocks the proteolytic processing of cathepsins B, L and D, resulting in accumulation of pro-cathepsins B, L and D in the lysosomes.

### Abnormal accumulation of pro-cathepsins in the lysosomes results in severe lysosomal dysfunction

**Figure 6:**
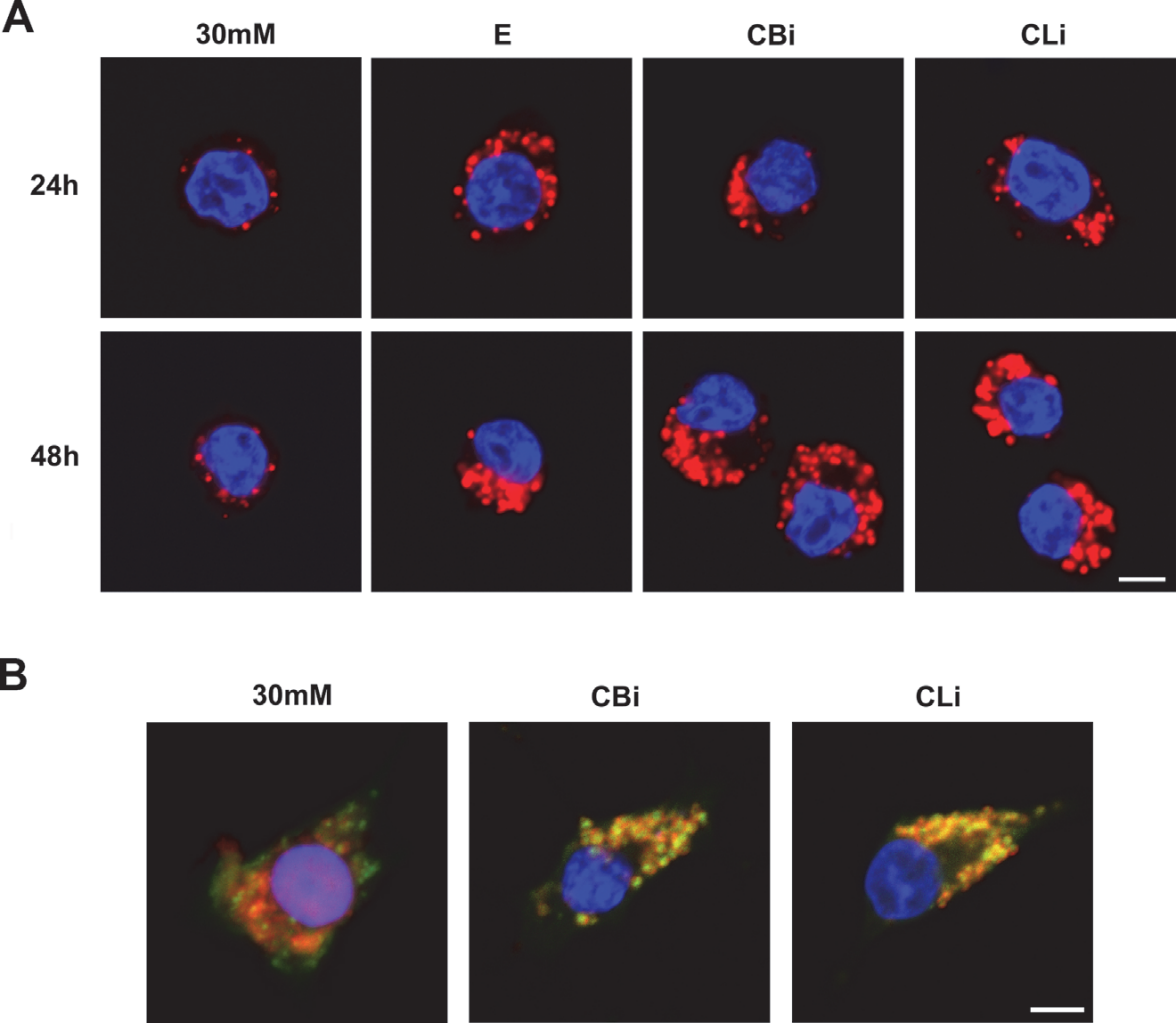
Inhibition of cathepsins B and L causes severe lysosomal dysfunction. (A) Lysotracker staining was performed in INS-1 cells cultured in 30 mM glucose for 24 or 48 hr, treated daily with E64d (E, 20 μg/mL), cathepsin B inhibitor (CBi, 20 μM), and cathepsin L inhibitor (CLi, 20 μM). Nuclei were stained with Hoechst 33342 dye. (B) INS-1 cells were transfected mRFP-GFP-LC3 construct and then treated with cathepsin B and L inhibitors in 30 mM glucose for 48 hr. 30 mM glucose medium alone increased red and yellow puncta (autolysosome, left panel), whereas treatment with cathepsin inhibitors increased only yellow puncta (autophagosome, middle and right panel). The scale bar represents 5 μm.

Interestingly, cathepsin inhibition by treatment with cathepsin B and L inhibitors resulted in enlargement of the lysosomes in a time-dependent manner compared with the control (Fig. 6A). It was next tested whether the enlarged lysosomes had the normal ability to form autolysosomes with autophagosomes, using mRFP and GFP tandem fluorescent-tagged LC3. mRFP-GFP-LC3 shows both GFP and mRFP fluorescence in the autophagosome before fusion with the lysosome. However, the GFP signal is quenched in acidic lysosomal conditions [54,55] whereas the mRFP-LC3 can be readily detected in autolysosomes with more stable fluorescence [56]. Therefore, the mRFP-GFP-LC3 tandem construct is a useful tool to trace the maturation process of autophagosomes into autolysosomes, by labeling the autophagosomes and autolysosomes in yellow and red, respectively [57]. Our results showed that only the yellow puncta were increased after treatment without an increase in the red puncta, indicating that the cathepsin B and L inhibitors blocked the maturation of autophagosomes into autolysosomes (Fig. 6B).

**Figure 7:**
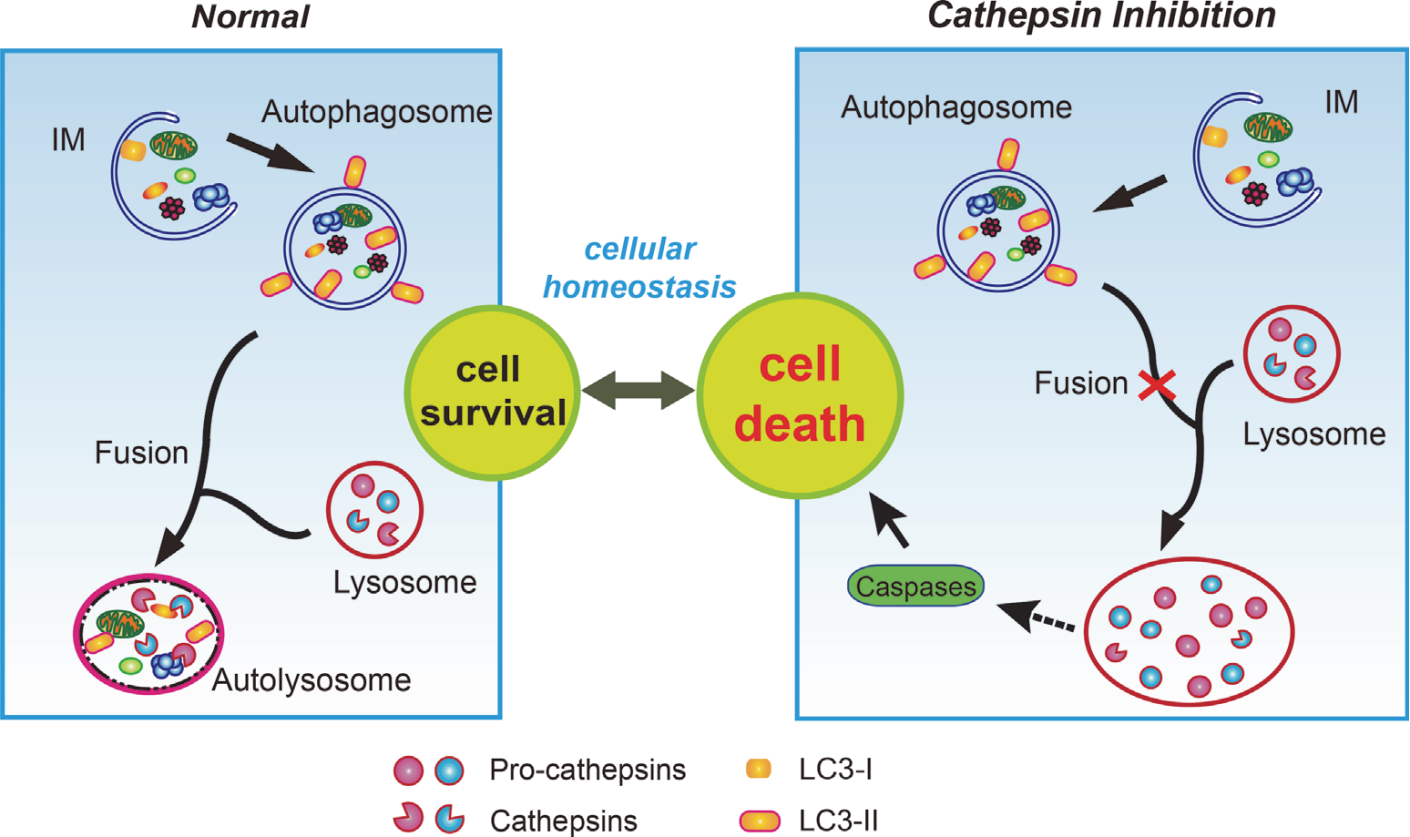
Lysosomal dysfunction by inhibition of cathepsins B and L causes cell death. In the macroautophagy pathway, impaired organelles are enclosed by a phagophore or isolation-membrane (IM), expansion of which gives rise to the autophagosome, a double-membrane vacuole that engulfs cellular components. Subsequently, the autophagosome fuses with lysosomes, in which lysosomal cathepsins, i.e. cathepsin B, D, L, etc. play a key role to allow normal function of the lysosome. Fusion of autophagosomes with lysosomes forms autolysosomes, playing a role in the degradation of cytoplasmic organelles. Inhibition of cathepsins B and L resulted in the accumulation of abnormal unprocessed cathepsins (pro-cathepsins) in the lysosomes. Abnormal accumulation of pro-cathepsins in the lysosomes leads to impaired autophagic process, especially fusion with autophagosomes, resulting in enlarged lysosomes. This lysosomal dysfunction indicates the phenomenon manifesting in lysosomal storage, finally inducing cell death by the activation of caspases.

Our results suggest that the impaired processing of cathepsins following the inhibition of cathepsins B and L leads to the accumulation of immature forms of cathepsins. Failure of cathepsin processing will prevent lysosomal degradation, resulting in enlargement of the lysosomes. Consequent impaired maturation of the autophagosome with lysosomes which may be responsible for the enhanced caspase-dependent apoptosis of pancreatic β-cells when exposed to high glucose (Fig. 7).

## Discussion

In our previous study, we showed that the chronic exposure of INS-1 cells and rat islets to high concentration of glucose induces autophagy as a protective mechanism [35]. In this study, pharmacological and genetic studies targeting lysosomal cathepsins demonstrated that the cysteine cathepsins, but not aspartic cathepsins, play a protective role in INS-1 cells and islets under glucotoxicity (Figs. 1 and 2). This suggests that the cysteine cathepsins may play an important role in the balance between cell death and survival in pancreatic β-cells under hyperglycemic conditions.

A normal lysosomal process is crucial for the clearance of long-lived proteins and impaired organelles via autophagic degradation. The process of protein degradation and organelle turnover is required for cell survival. Dysfunction of this process can result in abnormal cell growth or cell death, leading to various pathological conditions [58]. Recently, many studies have shown that the impairment of autophagy in pancreatitis is caused by defective lysosomal degradation [59–62]. These findings indicate that cathepsin processing is altered in pancreatitis, notably indicated by a decrease in the amount of mature cathepsins and an accumulation of immature pro-cathepsin [60–62]. Likewise, the defective processing of cathepsins causes lysosomal dysfunction in pancreatitis [61]. This lysosomal dysfunction leads to an impairment of autophagy, inflammation, and cell death, all of which are the known features of pancreatitis. Other studies reported that deficiency of lysosomal enzymes induces the impairment of autolysosome formation, leading to the accumulation of unprocessed substrates in the lysosomes. This has also been implicated in neurodegeneration, such as Alzheimer’s, Parkinson’s and Huntington’s diseases [63,64]. Failure of protein degradation due to lysosomal dysfunction mediates neuronal cell death [65]. Therefore, autophagy is relevant to cell survival by manipulating lysosomal function through cathepsins and their processing of lysosomal enzymes.

Lysosomes include various types of enzymes such as peptidases, phosphatases, and proteases. Among these enzymes, cathepsins are a major class of lysosomal protease, cleaved from pro-cathepsins and activated in the lysosomes. The cathepsins function optimally at low pH. In this study, the role of lysosomal cathepsins in pancreatic β-cell death was assessed upon inhibition of autophagy using lysosomal protease inhibitors (Fig. 3). Cysteine lysosomal proteases inhibitors triggered caspase-dependent cell death (Figs. 1 and 2), and the specific inhibition of cathepsin B and L using siRNAs enhanced apoptosis in high glucose conditions compared to normal glucose (Fig. 4).

Our data indicated that the pharmacological inhibition of cathepsins B and L blocks cathepsin processing (Fig. 5). Another study found abnormal function of the lysosomal enzymes to be present in models of pancreatitis [66]. Impaired processing of cathepsin was observed in experimental pancreatitis. As mentioned earlier, many studies have indicated that the accumulation of pro-cathepsins was caused by a blockade of cathepsin processing [60–62]. Here, interestingly, the inhibition of cathepsins B and L failed to proceed to mature forms not only of themselves, but also of other cysteine and aspartic proteases. Previous studies reported cathepsins B and L to be processed by cathepsin D [44,45]. Accordingly, we suggest that cathepsin maturation is involved in the cleavage and maturation of other cysteine and aspartic proteases.

In addition, accumulation of LC3-II and enlarged lysosomes were indicative of impaired lysosomal degradation caused by cysteine cathepsin inhibitors. Our results indicated that the inhibition of cathepsins B and L leads to the impairment of autophagy not only by decreased degradation activity, but also by inhibiting the processing of cathepsins and decreasing the levels of lysosomal membrane protein (Fig. 6).

Localization of the pro-cathepsins and cathepsins following the inhibition of cathepsins B and L was predominantly restricted to the lysosomes (Fig. 5E and 5F). Accumulation of undegraded and unprocessed cathepsins induces the malfunction of lysosomes, causing lysosomal storage disorders [67,68]. Enlarged and swollen morphology of the lysosome was seen when cathepsin B and L were inhibited. A similar effect was shown with mucolipidosis (ML-II), a deficiency of GlcNAc-phosphotransferase which mediates the mannose-6-phosphate recognition signal on lysosomal enzymes [69]. Proliferated lysosomes were observed to be filled with undigested substrates, suggesting impairment of autophagy [69,70]. These results indicate that defective lysosomal hydrolases and acidification lead to the disruption of lysosomal maturation and induction of lysosomal storage. In our study, the inhibition of lysosomal cysteine cathepsins in pancreatic β-cells led to enlarged lysosomes, implying defective lysosomal function. Defective lysosomal function is also related to impaired autophagic vesicle turnover. The mRFP-GFP-LC3 tandem construct is a useful tool to monitor the various stages of autophagic vesicle turnover, including fusion of autophagosomes with lysosomes and formation of an autolysosome. Expression of both RFP and GFP fluorescence signals in the autophagosome yields a yellow punctate signal, while the autolysosome exhibits only an RFP signal due to efficient quenching of the GFP signal in the acidic autolysosome. A substantial increase in the yellow signals, but not RFP, was observed in the cathepsin B and L inhibitor-treated cells, demonstrating impaired autophagic progression (Fig. 6B). Collectively, these data suggest that the defects in the maturation of autolysosomes mediated by cathepsin inhibitors may be due to a blockade of the fusion between autophagosomes and lysosomes.

Proper function of the lysosomal compartment is crucial to preserve cellular homeostasis. In early endosomes, pro-cathepsins are cleaved into active cathepsins. This maturation of cathepsins by autocatalysis or by other proteases is followed by translocation to the lysosomes [47]. It is important to note that the accumulation of pro-cathepsins upon treatment with inhibitors was limited to the lysosomes, not but to the ER. Many studies reported ER stress to cause pancreatic β-cell death [14,17]. However, no changes in the ER stress markers such as ROS, NO, PERK, and XBP-1 were observed after treatment of β-cells with lysosomal protease inhibitors (data not shown). Failure to process the lysosomal cathepsins and dysfunction of the lysosome are closely related to apoptotic cell death [71,72]. It is likely that a blockade by the inhibition of lysosomal proteases causes "lysosomal stress" that induces apoptosis, by a mechanism which is unknown as of yet (Fig. 7). Interestingly, however, our preliminary results indicated that cathepsin B and L inhibitors induced JNK activation at 24 hr (Fig. 2D). Although further studies are needed to determine the role of JNK activation, it will be intriguing to investigate whether the activation of JNK might be involved in the cellular pathway in response to lysosomal stress in the future.

In summary, our findings suggest that the impairment of autophagy by inhibition of cathepsins B and L induces cell death through lysosomal dysfunction, which prevents the maturation of these proteases in the lysosomes. As the first deleterious effect, the inhibition of cathepsins can have a functional impact, such as accumulation of pro-cathepsins in pancreatic β-cells and concurrent inhibition of the processing of other cathepsins in the lysosomes. Second, malfunction of cathepsins causes the accumulation of undegraded substrates and disruption of lysosomal function, such as blocking fusion with autophagosomes. Finally, lysosomal dysfunction is associated with cell death, which has a detrimental effect in the pathogenesis of diseases. Therefore, mechanistic studies to provide an understanding of lysosomal function (system) will help to understand the pathogenesis in many diseases.
